# Unmasking Pandemic Echoes: An In-Depth Review of Long COVID’s Unabated Cardiovascular Consequences beyond 2020

**DOI:** 10.3390/diagnostics13213368

**Published:** 2023-11-02

**Authors:** Maria-Luiza Luchian, Julien Higny, Martin Benoit, Benoit Robaye, Yannick Berners, Jean-Philippe Henry, Benjamin Colle, Olivier Xhaët, Dominique Blommaert, Steven Droogmans, Andreea Iulia Motoc, Bernard Cosyns, Laurence Gabriel, Antoine Guedes, Fabian Demeure

**Affiliations:** 1 Department of Cardiology, Université Catholique de Louvain, CHU UCL Namur Site Godinne, Av. Dr. G. Thérasse, 1, 5530 Yvoir, Belgiumantoine.guedes@chuuclnamur.uclouvain.be (A.G.); fabian.demeure@chuuclnamur.uclouvain.be (F.D.); 2 Department of Cardiology, Centrum voor Hart-en Vaatziekten, Universitair Ziekenhuis Brussel, Vrije Universiteit Brussel (VUB), Laarbeeklaan 101, 1090 Brussels, Belgium

**Keywords:** Long COVID, myocardial injury, chronic inflammation, microthrombosis, residual dyspnoea, Long Haulers, SARS-CoV-2, coronavirus

## Abstract

At the beginning of 2020, coronavirus disease 2019 (COVID-19) emerged as a new pandemic, leading to a worldwide health crisis and overwhelming healthcare systems due to high numbers of hospital admissions, insufficient resources, and a lack of standardized therapeutic protocols. Multiple genetic variants of severe acute respiratory syndrome coronavirus 2 (SARS-CoV-2) have been detected since its first public declaration in 2020, some of them being considered variants of concern (VOCs) corresponding to several pandemic waves. Nevertheless, a growing number of COVID-19 patients are continuously discharged from hospitals, remaining symptomatic even months after their first episode of COVID-19 infection. Long COVID-19 or ‘post-acute COVID-19 syndrome’ emerged as the new pandemic, being characterized by a high variability of clinical manifestations ranging from cardiorespiratory and neurological symptoms such as chest pain, exertional dyspnoea or cognitive disturbance to psychological disturbances, e.g., depression, anxiety or sleep disturbance with a crucial impact on patients’ quality of life. Moreover, Long COVID is viewed as a new cardiovascular risk factor capable of modifying the trajectory of current and future cardiovascular diseases, altering the patients’ prognosis. Therefore, in this review we address the current definitions of Long COVID and its pathophysiology, with a focus on cardiovascular manifestations. Furthermore, we aim to review the mechanisms of acute and chronic cardiac injury and the variety of cardiovascular sequelae observed in recovered COVID-19 patients, in addition to the potential role of Long COVID clinics in the medical management of this new condition. We will further address the role of future research for a better understanding of the actual impact of Long COVID and future therapeutic directions.

## 1. Introduction

Since the outbreak at the end of 2019, coronavirus disease 2019 (COVID-19) continues to exert a significant impact worldwide. More than 700 million infections have been reported globally, and up to 10% of recovered patients have described the persistence of symptoms months after their first episode of COVID-19 infection, irrespective of the referred pandemic wave [1]. Moreover, some studies have reported the presence of recurrent symptoms in up to 87% of recovered COVID-19 patients [2]. This inconsistency in data regarding the prevalence of symptoms following an acute episode of COVID-19 is due to the ongoing evolution of the definitions used to explain the post-acute coronavirus disease or post-acute sequelae of severe acute respiratory syndrome coronavirus 2 (SARS-CoV-2) infection employed by different studies as well as to the diversity of research protocols, population characteristics or type of COVID-19 variant analyzed.

Post-acute coronavirus disease 19 or Long COVID consists of more than 200 symptoms, with a high discrepancy in the multitude of symptoms and the actual clinical examination data and, consequently, its medical management. The current Long COVID definition remains vague and difficult to use in clinical practice.

To date, post-acute coronavirus disease 19 or Long COVID includes a high variety of symptoms, for example, shortness of breath, fatigue, muscle weakness or brain fog, present after 3 months from the index event, with a minimum of 2 months duration and without specific criteria for an alternative diagnosis [2,3]. Earlier reports suggested a possible correlation between Long COVID and the severity of COVID-19 pneumonia; however, it was also observed in patients with mild forms of COVID-19 infections [4]. In a study comprising more than 800 patients, residual symptoms following an acute episode of COVID-19 were described in 29.6% of patients, mostly observed in female patients, without being associated to the severity of the initial episode [5]. Although several clinical and epidemiological factors have been linked to the presence of sequelae after acute COVID-19 pneumonia, the underlying causes of Long COVID remain to be determined.

In light of this, a recent study analyzed the plasma samples of 63 Long COVID patients, emphasizing the presence of three SARS-CoV-2 antigens months after the acute episode, where the most frequently detected SARS-CoV-2 antigen was the spike [6]. The above-mentioned study sheds some light on potential causes of long-lasting symptoms in recovered COVID-19 patients by outlining the possibility of a latent active virus reservoir.

Nowadays, it is acknowledged that COVID-19 is more likely a multisystemic disease, with several anatomic sites responsible for virus replication [7] explaining the heterogeneity of symptoms both during the acute and chronic phases of COVID-19 pneumonia. The presence of the SARS-CoV-2 spike is known to alter pericytes, endothelial cells and the blood–brain barrier, promoting microthrombosis, heightened by local inflammation processes, and therefore acting as the pathophysiological substrate for the long-lasting symptoms in a significant proportion of patients [8,9]. Another potential cause related to the presence of Long COVID is the ongoing low-grade inflammation dysregulations in terms of the neutrophils count, neutrophil to lymphocyte ratio, C-reactive protein and fibrinogen [10], which were observed in several cohorts of patients recovering from the acute infection and still presenting residual symptoms.

Despite the newly emerging data on the mechanisms attributed to various acute cardiovascular and respiratory complications, during the index COVID-19 event, the amount of information on its long-term impact and Long COVID onset remains insufficient. In a post-COVID-19 pandemic era where the health and economic sectors are still recovering due to the redistribution of resources, the exact role of Long COVID programs and clinics remains debatable. Therefore, understanding the multisystemic effects of COVID-19 pneumonia and how to prevent them remains the cornerstone for a proper and timely medical treatment strategy to improve patients’ quality of life.

The aim of the present review is to assess the long-term consequences of SARS-CoV-2 infection in discharged patients, with a focus on cardiovascular complications. Furthermore, we will address the role of future research for a more in-depth evaluation of the actual impact of post-acute COVID-19 syndrome and, subsequently, potential therapies, including the role of Long COVID multidisciplinary clinics in this new clinical setting.

An extensive review of the current literature focused on COVID-19, published in English and indexed in Medline (PubMed), was conducted for the present article. The following key search words were included but were not limited to: COVID-19 residual symptoms, Long COVID, Long Haulers, post-acute COVID-19 syndrome, COVID-19 sequelae, COVID-19 myocardial injury, COVID-19 chronic fatigue, COVID-19 cardiac arrhythmias, COVID-19 neurological injury, COVID-19 residual dyspnoea, COVID-19 postural orthostatic tachycardia, heart failure and COVID-19, cardiomyopathy and COVID-19.

## 2. Post-Acute COVID-19 Syndrome—Mechanisms Hypothesis

### 2.1. COVID-19 Virus Strain and Vaccination

A growing number of patients are continuously recovering from SARS-CoV-2 infection, remaining symptomatic weeks or months after the initial episode, having a crucial impact on their quality of life and centered by the inability to return to the same health level as the one before the COVID-19 pneumonia, with significant reverberations across healthcare and economical systems worldwide. Factors linked to developing Long COVID are characterized by high diversity, from the health status of the patient, gender or age to the type of viral strain involved in the acute infectious process or vaccination status. Recent studies have emphasized major differences in terms of the concerned virus variant of the acute episode and the Long COVID susceptibleness. A case–control observational study with more than 90,000 patients showed a higher incidence of Long COVID cases within the COVID-19 population infected with the Delta variant than in patients with the Omicron variant, irrespective of the vaccination period [11]. Furthermore, patients with historical variant and higher COVID-19 disease severity were more likely to develop Long COVID, as opposed to patients infected with other variants such as the Alpha, Delta or Omicron variant or with less severe cases [12]. Although several reports attempted to diminish the effects on the study’s analysis of external factors such as the number of cases infected with a certain variant for a designed COVID-19 pandemic period, comorbidities, type of hospitalization or vaccination status, one must consider the high variability of the study population’s characteristics and the research methodology. Further, the accessibility to vaccination in addition to the medical treatment strategies used along the COVID-19 pandemic’s waves varied not only within different geographical areas but also within the same geographical region.

Patients infected with the historical variants were older and had more comorbidities, having an increased susceptibility to potential acute COVID-19 complications and subsequently to a higher number of long-lasting symptoms in comparison to COVID-19 patients infected by a more recent virus variant [12]. Additionally, one of the most common symptoms observed in the Long COVID population, dyspnoea, was highly expressed in patients recovering from the first waves [12]. In a study including 739 COVID-19 patients, 229 patients developed Long COVID, predominantly unvaccinated patients [13]. Moreover, having a COVID-19 vaccination booster was associated with a protective effect against Long COVID [13]. These findings are further strengthened by the results of an extensive survey including more than 300,000 participants with a sample analysis of 28,356 COVID-19-positive patients having at least one dose of COVID-19 vaccine [14]. Almost a quarter of the study’s population developed at least one symptom belonging to the Long COVID clinical spectrum during the follow-up [14]. Moreover, the same study showed that the presence of at least one dose of vaccine reduced the odds of developing Long COVID by 12.8%; furthermore, it was linked to a decreased risk of activity-limiting Long COVID, thus reducing the effects of Long COVID on the patients’ quality of life [14]. Hence, vaccination might induce a ‘reset mechanism’ on the dysregulated immune process due the acute SARS-CoV-2 infection, followed by the annihilation of any residual viral reservoir which may contribute to chronic inflammation [14]. Therefore, a possible substrate for future therapies in patients with residual symptoms after a viral infection might be represented by the modulation of the immune response by vaccination.

On the other hand, the results of the current study revealed no significant differences between the type of vaccine strategy or sociodemographic characteristics such as age, gender or ethnicity and the risk of Long COVID occurrence [14].

Although a recent meta-analysis comprising more than 6,000,000 patients showed no beneficial effect of vaccination after developing Long COVID symptoms, it outlined the same positive outcome of COVID-19 vaccination on reducing the severity of diseases and the Long COVID incidence [15]. Nevertheless, further prospective studies are necessary to confirm these results, especially in vaccinated patients.

### 2.2. Inflammation and Virus Reservoir—Potential Substrates for Long COVID

Several hypotheses on Long COVID substrates have been issued, ranging from multiple virus reservoirs, inflammation aberrations and coagulopathy to autoimmunity and multiple virus infections as underlying mechanisms for the persistence and the high discrepancy of symptoms in recovered COVID-19 patients. These processes may coexist, making it even more difficult to assess them in clinical practice in order to design therapeutic algorithms.

Vascular thromboinflammation may be one of the contributing mechanisms described in Long COVID [16]. The contribution of microvascular dysfunction is already acknowledged due to endothelial injury or platelet hyperreactivity and thrombocytosis, promoting the formation of circulating micro clots in the acute phase of SARS-CoV-2 infection, which is responsible for cardiovascular and respiratory complications in a significant number of patients [16,17,18,19,20]. Little is known if those mechanisms also persist during the post-acute COVID-19 period. Subsequently, the current data on potential pathophysiological mechanisms involving the immune system, inflammation and coagulation cascade in the onset of Long COVID symptomatology remain limited and conflicting. Most of the reported anomalies interfering with coagulation and inflammation processes were described in patients with an acute episode of COVID-19 pneumonia, as previously mentioned [7,9,20,21,22,23], being associated with worse prognosis, including higher rates of in-hospital mortality. Emerging from the previous data on the modulating effect of COVID-19 infection on the endothelial cells’ functions, researchers stipulated the ongoing procoagulant activity in convalescent patients as the cadre for Long COVID occurrence. A study consisting of 50 convalescent COVID-19 patients, predominantly hospitalized due to the severity of the acute SARS-CoV-2 pneumonia, showed coagulation plasma abnormalities, such as increased levels of plasma FVIII factor and endothelial cell activators including thrombin generation potential, when compared with a COVID-19-negative control group after 10 weeks following the acute episode [24]. Moreover, some studies reported the presence of persistent immunological disturbances in convalescent COVID-19 patients, from repressed levels of cortisol partially induced by the steroid therapy administered in the acute phase of the disease to reactivation of latent viral diseases, Epstein–Barr virus (EBV) or cytomegalovirus (CMV) [25], delineating potential treatment pathways for post-acute COVID-19 syndrome. Currently, there are several ongoing studies addressing various therapeutic regimens to treat Long COVID patients, such as Stimulate-ICP: Symptoms, Trajectory, Inequalities and Management: Understanding Long COVID to Address and Transform Existing Integrated Care Pathways, a multi-center randomized trial involving antihistamines (e.g., loratadine and famotidine), colchicine and rivaroxaban in Long COVID patients with chronic fatigue as the dominant symptom but not limited to it [26]. Other studies are focused on antiviral therapy, e.g., nirmatrelvir/ritonavir, in highly symptomatic Long COVID patients, with results to be published [27]. A simple search showed more than 150 ongoing registered studies on Long COVID. Chronic viral coinfections such as CMV, EBV or human immunodeficiency virus may be linked to some variants of post-acute COVID-19 syndrome phenotypes, consisting predominantly in neurological and gastrointestinal symptoms [28,29]. These results may further influence therapeutic decisions when it comes to medical management of Long COVID patients.

Persistence of virus reservoirs is an emerging hypothesis, contoured in autopsy and biopsy COVID-19 studies that demonstrated the presence of SARS-CoV-2 in PCR tissue analysis and tissue culture from various anatomic sites including stool samples [30]. Several reports indicated the presence of post-acute COVID-19 syndrome in patients with persistent viral antigens. A study conducted by Zollner A. et al. involving patients with inflammatory bowel disease showed SARS-CoV-2 ribonucleic acid in the gut mucosa at approximately 7 months after mild acute COVID-19, being further linked to the presence of Long COVID symptoms [31]. Contrastingly, the presence of long-lasting symptoms was not significantly correlated to the severity of the acute COVID-19 episode, immunosuppressive treatment or gut inflammation [31], hence strengthening the hypothesis of a virus reservoir as a substrate for Long COVID. Patients with residual symptoms exhibit chronic inflammation patterns after acute infection with persistent activated innate immune cells, diminished activity of naive T and B cells and elevated interferon type I IFN (IFN-β) and type III IFN (IFN-λ1) after the infection, in contrast to fully recovered COVID-19 patients or even to non-exposed patients, thus highlighting the abnormalities within the immune system at different time points in the evolution of the disease, driven by the autoimmunity and antigenic cross-reactivity [32].

Moreover, one key element supporting the Long COVID multitude of mechanisms hypothesis is the presence of gut microbiota disturbances in convalescent COVID-19 patients.

Two principal and opposite conditions were observed: an increase in opportunistic pathogens and a loss of commensal bacteria [33,34,35,36]. Supporting this statement, several opportunistic pathogens, including unclassified Escherichia, Intestinibacter bartlettii, Clostridium aldenense, inflammation-related pathogens including Clostridium bolteae and Flavonifractor plautii and a COVID-19-severity-related pathogen (Clostridium ramosum) were identified in convalescent COVID-19 patients, whereas a depletion of beneficial commensal bacteria was observed as well, being further associated with recurrent symptoms, e.g., fatigue and muscle weakness [33,35,36]. Although the role of gut microbiota and recovery after COVID-19 have been extensively studied, there are still missing data on its real impact on Long COVID.

Hence, the presence of chronic inflammation due to the acute infectious process and the persistence of virus reservoirs in different anatomical sites, promoting unbalanced gut microbiota, may represent another important aspect to be considered for future strategies on prevention and Long COVID treatment.

## 3. The Clinical Spectrum of Long COVID

**Figure 1:**
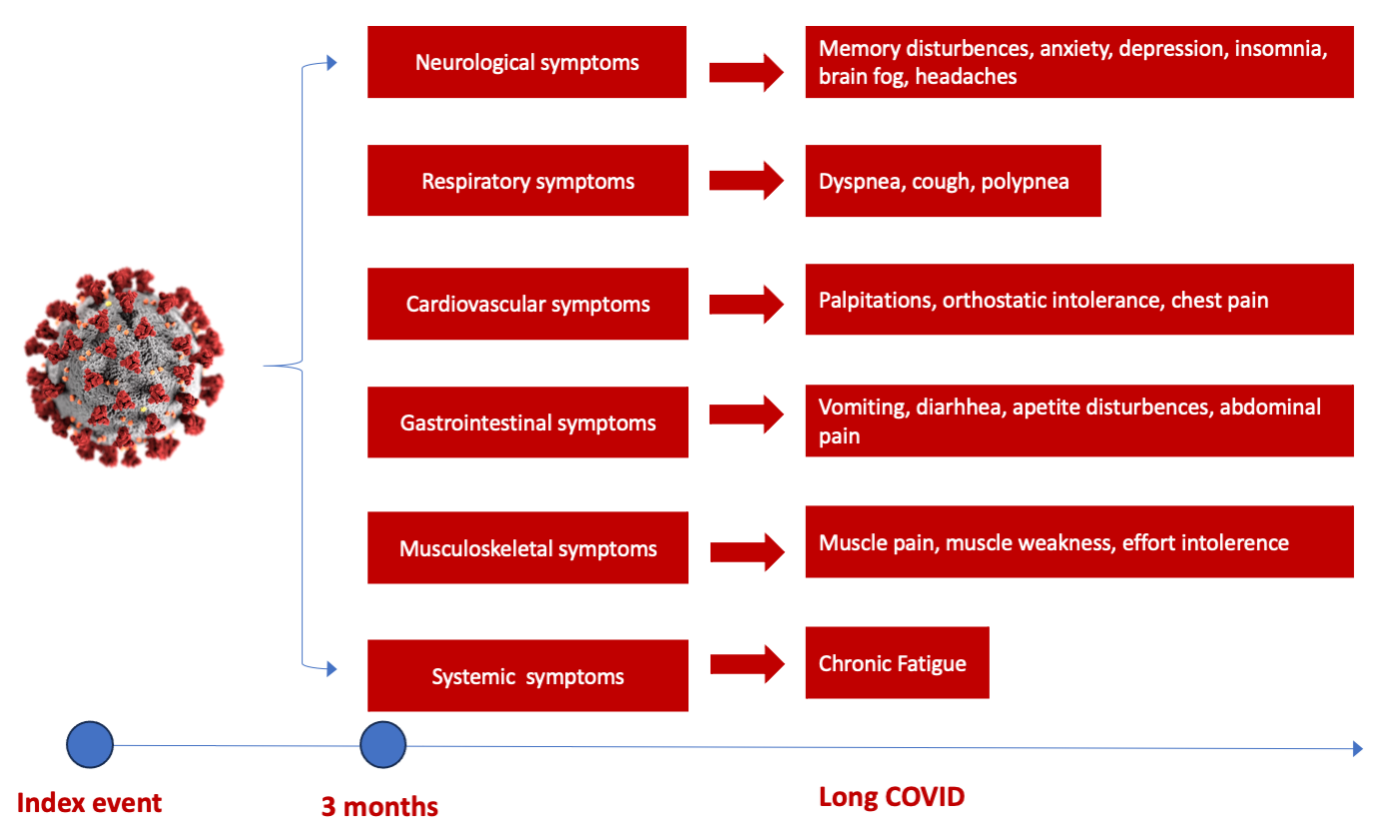
The spectrum of Long COVID symptoms according to the organ involved. The most commonly reported neurological and cognitive symptoms are brain fog, sleep and memory disturbances and cognitive disturbance, followed by respiratory and cardiovascular symptoms such as palpitations, chest pain and dyspnoea, gastrointestinal symptoms (weight loss, vomiting, diarrhea) and musculoskeletal symptoms (muscle pain or weakness), dominated by chronic fatigue [38].

Several studies have shown a discrepancy in Long COVID prevalence around the world, with the highest number of reported cases in Asia, followed by Europe and America [37]. Moreover, it appears there is a small difference between hospitalized and non-hospitalized COVID-19 patients at risk of developing Long COVID. The highest risk of mortality and post-acute COVID-19 sequelae was observed in hospitalized patients in intensive care units, followed by normal ward hospitalization and ambulatory treated patients [38]. The most frequent sequelae reported in several studies are attributed to the cardiorespiratory system, followed by hematological and neurological abnormalities [38]. Initial symptoms of Long COVID predominantly encompass fatigue and dyspnoea in up to 50% of patients, followed by muscle weakness, palpitations, thoracic pain or anxiety and cognitive impairment to various degrees [38,39] (Figure 1). Concentration difficulties or compromised sleep to anxiety and depression induced by potential brain hypometabolism, cerebral cortex hypoperfusion or brain structure and functional connectivity disruptions are some of the neurological sequelae that COVID-19 survivors experience after an acute episode, leading to accentuated fatigue and an inability to continue normal activity [37,38,40,41].

Some studies were able to distinguish dissimilarities in terms of the debut of Long COVID symptoms and their duration. According to a recent meta-analysis of 63 studies, in the first 3 to 6 months, in addition to fatigue and dyspnoea, convalescent COVID-19 patients exhibited various levels of concentration difficulties and sleep disturbances, whereas in the period 6 to 9 months, Long COVID patients mainly described effort intolerance [42]. After one year, the prevailing symptoms were fatigue, dyspnoea, sleep disorder, muscle pain or weakness [42].

Gender-specific differences were also outlined in various reports. However, it remains debatable whether women present a higher susceptibility to Long COVID than men [40,41,42,43].

## 4. Cardiac Injury and Long COVID

**Figure 2:**
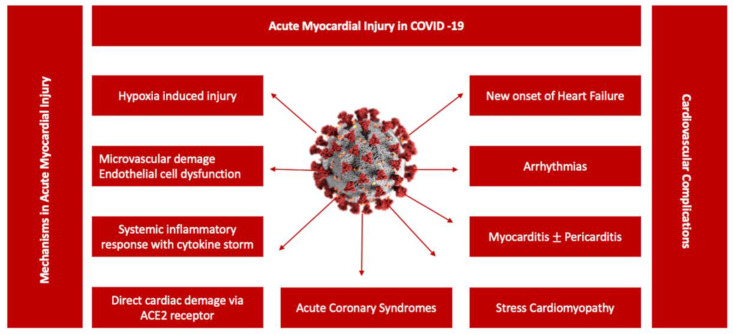
Potential mechanisms associated with acute myocardial injury in COVID-19. A central role in the pathogenesis of COVID-19 cardiovascular involvement is played by endothelial dysfunction due to a variety of pathophysiological mechanisms, from systemic inflammatory response syndrome including cytokine storm to hypoxia-induced injury or microvascular damage due to perfusion defect, translated in a diverse spectrum of clinical scenarios from acute myocarditis to stress cardiomyopathy [47].

Persistent cardiac injury after an acute episode of COVID-19 pneumonia continues to be an important research focus for clinicians, especially in COVID-19 survivors with long-lasting symptoms. The clinical significance of persistent elevation of cardiac biomarkers such as cardiac troponin (cTn) or NT-proBNP is not fully understood, irrespective of the chosen clinical topic. Nevertheless, cTn persistence without diagnostic criteria for specific cardiovascular diseases is associated with higher rates of major cardiovascular events (heart failure, stroke or infarction), including all-cause mortality [44]. Subsequent studies showed a significant liaison between cTn levels suggestive of myocardial injury and poor outcomes in hospitalized COVID-19 patients [22,45,46,47] (Figure 2). However, its incremental value in Long COVID patients is insufficiently investigated. Different patterns of cardiac injury have been associated with mid- and long-term consequences following acute COVID-19 [48,49,50,51,52,53,54]. During convalescence, patients recovering from COVID-19 may exhibit a great variety of symptoms, as previously mentioned, that may be partially correlated to clinical and subclinical heart dysfunction, suggesting a possible background for Long COVID physiopathology. Cardiovascular imaging including cardiac magnetic resonance (CMR) and echocardiographic examinations represented the foundation of the majority of short-term follow-up studies focusing on the predictive value of acute cardiac injury in COVID-19. During the acute phase of COVID-19 pneumonia, the clinical manifestations of cardiac injury have included: acute cardiac ischemia, myopericarditis, acute onset of arrhythmias, stress cardiomyopathy or heart failure associated with various degrees of left ventricle function, global or segmental abnormalities, right ventricle dysfunction, diastolic dysfunction or pericardial effusion [48,55,56,57,58,59,60,61]. Moreover, the highest prevalence of major cardiovascular events including mortality was observed in COVID-19 patients with elevated cardiac biomarkers and functional cardiac imaging abnormalities [48,62]. Furthermore, CMR abnormalities varying from ischemic to non-ischemic late gadolinium enhancement patterns to pericardial enhancement were reported from 26% up to 60% of recovered COVID-19 patients, with a significant difference between hospitalized versus non-hospitalized patients [63]. In patients without previous comorbidities and a mild form of COVID-19 pneumonia, cardiac involvement on CMR studies remained exceptional, without a relation to the ongoing symptoms [64,65,66]. Moreover, when compared to the healthy control group, no significant structural or functional cardiac abnormalities including strain, perfusion or advanced tissue characteristic differences were found [65,66]. However, these data remain contradictory due to population heterogeneity, study protocols and methodology. For example, some CMR studies described higher myocardial native T1 mapping values in symptomatic Long COVID patients at one-year follow-up, suggestive of chronic cardiac inflammation, without signs of a specific cardiac structural disease or detectable cTn [67]. In a consecutive cohort of 121 patients with a history of COVID-19 pneumonia, almost half of the study participants, predominantly those with more severe forms of pneumonia requiring hospitalization, presented non-ischemic cardiac injury [68]. However, according to the authors, further clinical evaluation may not be required, as these findings were common in COVID-19 survivors without a correlation to a specific symptom or a structural cardiac disease [67,69,70]. A more recent study attempted to better classify Long COVID patients based on the symptomatology, presence of chronic cardiac injury defined by the persistence of elevated cTn and NT-proBNP and CMR abnormalities during the acute episode and at 6- and 1-year follow-up [71]. In a cohort of 534 patients, 1% of patients had detected biomarkers at 6 months follow-up, whereas 19% of the patients with normal values of biomarkers presented various degrees of cardiac dysfunction, identified on CMR follow-up exams at 6 months [71]. The most recurrent CMR abnormalities described in Long COVID were reduced left ventricle and right ventricle ejection fraction in up to 21% of the patients and reduced left global longitudinal strain and T1 elevation, with similar findings being described in echocardiographic studies [71,72,73]. The residual symptoms such as fatigue, breathlessness or thoracic pain were predominantly reported by patients with cardiac dysfunction on CMR; however, there was no correlation between the presence of abnormal biomarkers, CMR findings and Long COVID symptomatology [71]. In 42% of the patients, a resolution of cardiac abnormalities was observed at one-year follow-up, with no correlation with the improvement of Long COVID symptoms [71]. Importantly, the onset of Long COVID was not linked to the type of medical management, hospitalization or ambulatory treatment. [71].

During the acute phase, the dominant cardiovascular clinical scenarios were represented by myocarditis, pericarditis, myocardial infarction and pulmonary embolism or tachyarrhythmias, whereas in the Long COVID population the typical scenarios are centered around arrythmias, systolic and diastolic left ventricle function, new onset or aggravation of the pre-existing heart failure and ischemic and non-ischemic cardiomyopathies including silent myocarditis [74]. The substrate of the acute cardiac dysfunction described during acute COVID-19 pneumonia was attributed especially to the myocardial injury induced by direct virus action in combination with other mechanisms such as abnormalities in the host immune response, dyselectrolytemia, hypoxia-induced injury, microvascular damage due to a perfusion defect exacerbated in patients with advanced age, obesity and previous cardiac comorbidities [75,76,77]. Earlier reports showed the presence of increased levels of inflammatory biomarkers such as interleukin 6, C-reactive protein, fibrinogen or procalcitonin in patients with COVID-19, being associated with a worse outcome [78]. Dysfunction of the endothelial cells, which express the angiotensin-converting enzyme 2 (ACE 2) receptor, known as the entry site for SARS-CoV-2, triggers an overexaggerated immune response, with an extensive production of proinflammatory and prothrombotic factors [24,79,80]. Further, this induces coagulopathies, imbalanced prothrombotic response and microthrombus formation leading to the multisystemic consequences seen in clinical practice [79,80]. One interesting aspect of the multisystemic mechanistic approach of the acute and long-term outcomes of SARS-CoV-2 infection is represented by the capacity to release autoantibodies against cardiac antigens, which is a process triggered by uncontrolled inflammation [81]. The presence of anti-cardiac autoantibodies may be responsible to some extent for the long-term consequences of COVID-19. In one study of 104 hospitalized COVID-19 patients, 68% (71 patients) had anti-cardiac autoantibodies, irrespective of the patients’ comorbidities or age [81]. Moreover, several reports mentioned the presence of antiphospholipid antibodies, especially during the acute phase of COVID-19 pneumonia, which are already acknowledged as important actors of immune-mediated thrombosis [82]. These results should be cautiously interpreted, considering that the presence of specific antibodies was not assessed before COVID-19.

Having this multisystemic approach when analyzing COVID-19 phenotypes, it seems plausible that after an acute episode of COVID-19 the duration of an increased risk of cardiovascular events may be prolonged. Within the US Veterans Health Administration study, the investigators showed a prolonged risk of cardiovascular events, especially acute coronary syndromes (hazard ratio, 1.63 [95% CI, 1.51–1.75]) and stroke (hazard ratio, 1.52 [95% CI, 1.43–1.62]) surpassing 30 days [83]. Moreover, over 2 years of follow-up, the risk of all-cause health consequences including cardiovascular sequelae in non-hospitalized COVID-19 patients remained increased for 31% when compared to patients without a history of COVID-19 infections, suggesting that irrespective of the severity of the disease, COVID-19 exerts a long-term effect on health status, including cardiovascular health [84]. Furthermore, the same study reported an augmented risk of all-cause mortality and hospitalization at 2 years follow-up in the COVID-19 hospitalized group when compared to patients without COVID-19 infection or non-hospitalized COVID-19 patients [84]. However, during the period of 2 years after the first episode of COVID-19, the risk of organ damage, hospitalization and mortality decreases, especially in non-hospitalized COVID-19 patients, whereas in hospitalized COVID-19 patients the risk of death, hospitalization and organ sequelae including the cardiovascular, hematological or respiratory system remained significantly elevated, showing the difficult and enduring road to recovery [84].

Whether the severity of COVID-19 pneumonia plays a key role in developing long-term cardiac sequelae in COVID-19 survivors with residual symptoms, more prospective studies with a longer follow-up duration are mandatory.

## 5. Different Cardiac Clinical Scenarios in Long COVID

### 5.1. Postural Orthostatic Tachycardia Syndrome

Palpitations and chest pain remain one of the most frequent complaints seen in Long COVID patients. As previously mentioned, acute cardiac damage was frequently described during COVID-19 pneumonia, being associated with a poor prognosis [85,86]; however, its relation to residual symptoms is still argued. Several reports have emphasized a high incidence of orthostatic intolerance (OI), including orthostatic hypotension (OH), defined as a reduction in blood pressure of at least 20 mm Hg of systolic blood pressure or 10 mm Hg of diastolic blood pressure within 3 min of standing, and postural orthostatic tachycardia syndrome (POTS), an increase in heart rate of more than 30 bpm within 10 min of standing or during a tilt test in Long COVID patients, probably due to hypovolemia or deconditioning after prolonged bed rest [87,88]. Abnormal autonomic nervous system response to orthostatism, in addition to exaggerated levels of epinephrine and norepinephrine, contributes to the pathophysiological spectrum of mechanisms in OI that may be further accentuated by multiple factors such as excessive venous pooling in the lower extremities, volume dysregulations, autoimmunity and hyperadrenergic status [89]. Earlier reports emphasized a downregulation of renin activity and abnormal levels of angiotensin II in POTS patients [90,91,92,93]. This is further intensified in COVID-19 infection, as previous studies reported [94,95]. The renin angiotensin aldosterone system (RAAS) imbalance triggered by SARS-CoV-2 infection may have a fundamental role in Long-COVID-related POTS [91,95,96,97]. Moreover, SARS-CoV-2 may damage the extracardiac postganglionic sympathetic nervous system, promoting dysautonomia and leading to POTS [91,92,98]. Other mechanisms such as autoimmunity or chronic inflammation have also been attributed to COVID-19 long-term sequelae including OI onset [99]. The true prevalence of OI in Long COVID patients is difficult to determine, considering it still remains an underdiagnosed syndrome. Yet, several reports described symptoms consistent with OI in up to 41% of recovered COVID-19 patients [100,101]. Potential limitations of these reports are represented by the retrospective design, with missing data prior to COVID-19 infection and a limited number of patients [102]. However, most of these reports are based on symptoms evaluation after COVID-19, implying newly diagnosed conditions. One report described a prevalence of 38% of OI in Long COVID patients [103]. These data are being supported by previous research on Long COVID and OI, suggesting autonomic dysfunction, which is known as a common finding during patients’ recovery after bacterial or viral infections [87,104]. Although several standard factors such as deconditioning, presence of venous insufficiency or hypovolemia may aggravate the symptomatology, the presence of OI was also observed in COVID-19 patients that did not require prolonged hospitalization and were apparently healthy before COVID-19 pneumonia [105,106].

As there is no specific treatment for POTS, current lifestyle measurements include: adequate hydration, specific exercise training and various pharmacotherapies, depending on POTS subtypes such as ivabradine or β-blockers, in combination with compression stockings when tachycardia is the dominant symptom to midodrine for persistent OH due to venous polling or fludrocortisone if hypovolemia and orthostatic intolerance are associated [87,107].

### 5.2. Arrhythmias and COVID-19

During the acute phase of COVID-19 pneumonia, acute cardiac injury was commonly described, especially in critically ill patients. Several clinical scenarios have been outlined from acute onset of arrhythmia, especially atrial fibrillation, to myocardial infarction or myopericarditis [18,47]. The majority of these reports defined acute cardiac injury based on the values of a biomarkers assessment, predominantly upon admission and cardiovascular imaging assessment, especially echocardiography or CMR, during cardiovascular complications. Considering the abnormalities in the coagulation and inflammation processes triggered by the viral action as the background for the high diversity of complications observed during an acute episode and also the conglomeration of symptoms reported during post-COVID-19, it is no surprise that different degrees of myocardial strain imaging deformation abnormalities may be identified in COVID-19 patients [108]. COVID-19 patients with persistent dyspnoea and exercise intolerance after an acute episode had impaired left atrium (LA) strain functions, particularly LA reservoir strain and LA stiffness suggesting LA myopathy, as indirect signs of diastolic dysfunction [72,108]. These changes in LA function are consistent with the onset and the severity of atrial fibrillation (AF) and the subsequent atrial fibrosis [108,109,110,111,112]. However, the spectrum of cardiac arrhythmias is characterized by a high diversity and complexity, from supraventricular arrhythmias, especially atrial fibrillation, to ventricular tachyarrhythmias, bradyarrhythmias (BAs) and conduction defects [113].

### 5.3. Bradyarrhythmia and Atrioventricular Blocks

During the acute phase, more than 10% of COVID-19 patients developed AF during hospitalization [114]. A similar number of patients developed ventricular arrhythmias [115]. The electrocardiogram (ECG) presentations seen in COVID-19 patients were predominantly represented by sinus tachycardia, followed by supraventricular tachycardia atrial fibrillation/flutter, ventricular tachycardia, QT prolongation and BA [116]. BA and atrioventricular blocks (AVBs) were less frequently observed in COVID-19 patients when compared to the prevalence of sinus tachycardia or supraventricular arrythmias [116]. BAs reported during the acute phase of the disease were mainly due to myocardial inflammation and endothelial injury in the context of a cytokine storm, hypoxia and electrolytes imbalance, all processes leading to the aggravation of pre-existing conduction abnormalities or the onset of new ECG changes [116,117]. Importantly, approximately 7.5% of myocardial cells express ACE 2 receptors [116]; therefore, direct virus action on myocardial cells leading to cardiac injury and consequently to the onset of arrythmias may represent one key element of the underlying pathophysiology. Moreover, several medications used during the first waves of the COVID-19 pandemic such as chloroquine, hydroxychloroquine and azithromycin may induce QT interval prolongation, leading to torsade de pointes [116,118]. The term viral channelopathy has recently emerged, explaining this subset of arrythmias occurring during viral diseases. Viruses are able to encode their own ion channels called viroporins in the host cell [119]. Consequently, some ion-channel-blocking drugs may demonstrate antiviral activity [119]. By understanding this new virus mechanistic concept, the medication used to treat viral diseases my contribute not only to virus annihilation but also to bypassing virus-induced channelopathies [119]. The host ion channels, especially the K^+^ and Ca^2+^ channels, participate at different stages of virus cycle; therefore, some dormant channelopathies may be exacerbated by viral infections [118]. Moreover, viral-modulated channel action may impact the cell contractility, inducing arrythmias, further enhanced by indirect factors, e.g., cytokine actions, endothelial hypoxia-induced injury or due to the treatment performed [118]. In this perspective, ongoing inflammation may act as the dominant substrate for long-term consequences of COVID-19 infection, where one key element of the chronic low-grade inflammation hypothesis is represented by the transforming growth factor beta (TGF-β) activity [120]. Activation of TGF-β is linked to inflammation, apoptosis and fibrosis, hence playing a crucial role in the acute and long-term effects of COVID-19 [120]. By modulating the cascade of signaling of TGF-β pathways, the deleterious effects may be circumvented. Irrespective of COVID-19 severity, the plasma levels of TGF-β, especially TGF-β1, were heightened in hospitalized COVID-19 patients, with an abnormal and uncontrolled immune response induced by the SARS-CoV-2 infection [121]. TGF-β1 dysregulation is associated with cardiac myofibroblast arrhythmogenicity [119,121] as a substrate for high-degree AVB in COVID-19 survivors [122]. Additionally, the presence of fibrosis facilitates re-entry circuits, which further triggers arrythmia genesis [113]. Nevertheless, data on arrhythmic events after the COVID-19 pandemic remain limited and divergent. Most reports on the prevalence of arrhythmic events are during acute infection, when the medical management may be encountering difficulties due to acute decompensation of other pathologies. During an acute episode of COVID-19 pneumonia, arrhythmic events were reported in approximately 5% to 10% of the patients [123]. In post-acute COVID-19 syndrome, the prevalence is difficult to establish due to limitations regarding study designs, including population characteristics, duration of the follow-up and patients lost to follow-up. In a Swedish cohort including more than 3000 patients with a history of severe COVID-19 infection, the incidence rates per 1000 persons-years of ventricular tachycardia, atrial fibrillation, other tachyarrhythmias and bradycardia/pacemaker implantation were 15.4, 78.4, 99.3 and 8.5, respectively, when compared to the general population, at 9 months follow-up [124]. A larger report including more than 600,000 COVID-19 survivors, without previous cardiovascular diseases prior to COVID-19 infection, showed a higher 12-month risk of arrythmias, especially atrial fibrillation and flutter (HR = 2.407 [2.296–2.523]), followed by tachycardia (HR = 1.682 [1.626–1.740]), ventricular arrhythmias (HR = 1.600 [1.535–1.668]) and bradycardia (HR = 1.599 [1.521–1.681]) when compared to the matched control group without a history of SARS-CoV-2 infection [125]. Although a trend in overestimation, the severity of cardiovascular outcomes in COVID-19 survivors was observed in smaller cohort studies; when compared to a population without documented COVID-19 infection, COVID-19 survivors have a higher risk of cardiovascular complications including mortality, irrespective of the severity of the acute episode [126,127]. Therefore, individualized medical management including referral to specific departments (electrophysiology, POTS clinics, heart failure clinics), especially in patients with persistent symptomatology following COVID-19 pneumonia, should be implemented.

### 5.4. Relative Bradycardia and COVID-19

Relative bradycardia (RB) is another phenomenon different from BA, being characterized by an abnormal response to high body temperature observed in various infectious diseases, including COVID-19 [128]. Several definitions have been proposed to characterize RB. In general, RB is defined by a less than 10 beats/minute rise in body temperature [128,129]. The prevalence of RB in the COVID-19 population is between 36% and 76% [128], depending on various factors from age, associated comorbidities like diabetes or use of antipyretic drugs to the release of inflammatory cytokines, increased vagal tone, direct virus effect on the myocardium and electrolyte disturbances, which are frequently described in COVID-19 pneumonia [129].

### 5.5. Heart Failure and COVID-19

As the central mechanisms for COVID-19 and cardiovascular involvement are represented by inflammation, heart failure (HF) and COVID-19 during the acute and post-acute phase may coexist.

Common risk factors for poor prognosis in both pathologies remain older age, obesity and diabetes [130,131,132]. Importantly, the absence of systematic HF follow-up during the COVID-19 pandemic due to restrictions and the fear of patients contracting COVID-19 pneumonia contributed to the severity of both conditions, acute cardiac injury induced by COVID-19 and HF [132,133]. Consequently, during the COVID-19 pandemic, the post-discharge mortality among HF patients was especially augmented due to insufficient access to specialized care [134]. In one study analyzing the incidence of new HF onset or HF worsening before and after lockdown during the first COVID-19 pandemic wave, the investigators showed a significant decrease in the number of new HF diagnoses or HF worsening when compared to the pre-pandemic period, raising concerns about HF undertreatment and potential long-term consequences following COVID-19 [135].

Considering all those factors, several reports outlined a 2% HF incidence in COVID-19 survivors [133,136]. A meta-analysis comprising a large population showed an additional risk of 90% developing HF after COVID-19 infection that rises with age and the presence of arterial hypertension [136].

Endothelial dysfunction, microvascular damage, ongoing inflammation and prothrombotic state following acute COVID-19 pneumonia may play a significant role in multiorgan dysfunction, including new-onset or aggravating a pre-existing HF [137,138]. Several cardiac abnormalities have been reported in post-acute follow-up COVID-19 studies, highlighting the importance of continuous evaluation. The presence of subclinical cardiac dysfunction was a common finding in those studies, focusing on COVID-19 survivors. Nevertheless, a current limitation encountered in numerous studies focusing on discharged COVID-19 patients was represented by insufficient data on cardiac function prior to or even during COVID-19, due to a lack of systematically transthoracic echocardiography evaluations or incomplete diagnosis work-up in order to reduce cross-infection of the healthcare professional or transmission to other patients. During acute setting of the disease, studies have shown a significant correlation between the presence of right ventricle (RV) dysfunction and the onset of major cardiovascular events and in-hospital mortality [139]. At follow-up, CMR studies showed the persistence of abnormalities with lower RV and LV ejection fractions and higher ventricle volumes when compared to healthy controls, suggesting potential cardiac dysfunction associated with Long COVID [50]. Although the majority of discharged patients presented normal LV function at 3 to 6 months, abnormal echocardiographic findings such as RV adverse remodeling with RV dilatation or biventricular dysfunction were predominantly observed in patients with acute cardiac events (e.g., pulmonary embolism) during hospitalization [140], suggesting a correlation between the severity of the acute infection and the mid- and long-term consequences.

In one study of 367 participants, 53% of discharged COVID-19 patients without known cardiovascular diseases or other significant comorbidities prior to COVID-19 and mild episodes of COVID-19 pneumonia, followed-up at 109 days and 329 days with echocardiography and CMR, developed cardiac symptoms after COVID-19, whereas 23% remained asymptomatic during the entire follow-up, and only 20% converted from symptomatic to asymptomatic status [67]. The echocardiographic evaluation showed preserved LV and RV systolic functions and low values of LV global longitudinal strain, though still in the normal range irrespective of the presence of residual symptoms [67]. Compared to asymptomatic patients, patients with Long COVID and cardiac symptoms showed higher myocardial native mapping values, suggestive of inflammation and pericardial enhancement, suggesting pericardial inflammatory involvement [67]. Although mapping values improved by the end of the follow-up, they showed a trend toward higher values when compared to asymptomatic patients or the ones who became asymptomatic during the follow-up, emphasizing persistent low-grade inflammation without the presence of ischemic or structural cardiac disease [67]. Although more than half of the study population had traceable levels of cardiac troponin, its presence did not correlate with the ongoing cardiac symptoms [67]. Additionally, structural heart disease, e.g., reduced LVEF or reduced RV ejection fraction or dilated cardiomyopathy, was rarely reported in this study [67]. In the same direction, a 10-week follow-up study of 139 healthcare workers with confirmed past SARS-CoV-2 infection showed 75% CMR abnormalities incidence in asymptomatic patients [141]. Moreover, the same report showed evidence of pericarditis or myocarditis patterns in up to 40% of cases following an acute episode of SARS-CoV-2 [141]. Although the presented study is limited by the small sample size, the value of the research comes from the exhaustive examinations of the patients, including CMR, ECG and laboratory exams. Nevertheless, some of the findings reported by those follow-up studies may be incidental, due to a lack of previous imaging data before COVID-19 infection.

Contrastingly, earlier reports focusing on myocardial injury defined by increased plasma levels of cardiac biomarkers, e.g., cardiac troponin, showed no significant echocardiographic differences between discharged COVID-19 patients with cardiac injury during hospitalization and patients with normal values of cardiac biomarkers [142]. Moreover, they suggested a full recovery of cardiac function and no evidence of cardiac dysfunction. Hence, data on cardiac dysfunction attributed to COVID-19 are conflicting. Small cohort studies tend to overestimate or to underestimate the actual cardiovascular impact of COVID-19. For example, in a large cohort of more than 1500 participants, CMR screening for myocardial abnormalities after COVID-19 showed an incidence of 2.3% clinical and subclinical myocarditis, with reduced left ventricular systolic function, pericardial inflammation and effusion, out of which 1.8% were asymptomatic during the follow-up [143]. The incomplete recovery with residual dysfunction and negative remodeling after COVID-19 may represent the framework for HF onset. The lack of prospective studies with a sufficient sample size makes it difficult to assess the true impact of COVID-19 on heart function. HF was a constant finding in patients with acute COVID-19, irrespective of the presence of other comorbidities [133,144]. One report consisting of 100 patients hospitalized with COVID-19 showed a normal echocardiogram only in 32% of the patients, whereas 16% presented LV diastolic dysfunction, 39% RV dilatation or dysfunction and 10% LV systolic dysfunction [145]. Subsequently, patients with COVID-19 are at a higher risk of HF exacerbations. New onset of HF was also reported in almost a quarter of COVID-19 patients, and it was heightened in patients with severe forms of COVID-19 admitted to intensive care units [144]. Acute cardiovascular events during COVID-19, e.g., pulmonary embolism leading to RV dysfunction, stress cardiomyopathy or acute myocarditis, may represent the framework for HF development, having a significant impact on survival and promoting long-lasting cardiac dysfunction attributed to partial recovery of COVID-19 survivors [144].

### 5.6. Coagulation Abnormalities and COVID-19

Several reports focusing on lung parenchyma abnormalities following an acute episode of SARS-CoV-2 infection emphasized increased lung density and glycolytic metabolic activity, advocating for increased inflammation and endothelial activation, leading to a procoagulability status [146]. Detected levels of D-dimer were reported in COVID-19 survivors even at 4 months after the acute episode [147]. In hospitalized patients, circulation of hyperreactive platelets may contribute to the hypercoagulability state that may persist even after the resolution of the acute episode [147]. Additionally, COVID-19 survivors presented high plasma viscosity and fibrin amyloids [146,147]. Those microthrombi express α2-antiplasmin, an enzyme that blocks proteolytic activity of plasmin and subsequently their degradation [146,147]. The morphofunctional abnormalities described in Long COVID patients on chest computed tomography follow-up studies coupled with functional respiratory tests included: fibrotic-like and non-fibrotic abnormalities, perfusion defects or areas of increased perfusion [148].

It is difficult to estimate the true prevalence of thrombotic events in Long COVID, as the majority of information is obtained from sporadic events or series of case reports. In the post-acute phase of COVID-19 pneumonia, the incidence of venous thromboembolic events was considered to be below 5% [149]. Other post-discharge reports described an incidence of combined thrombotic events (venous thromboembolism, stroke, pulmonary embolism, intracardiac thrombi) in up to 2.5%, whereas for venous thrombotic events it was 0.6% [150]. It remains high in the first 45 days after discharge and diminishes progressively [151]. In addition to the intrinsic factors, the susceptibility to developing thrombotic events is influenced by the standard risk factors from the severity of the disease, intensive care unit admission or duration of hospitalization to the comorbidities of the patients [149].

An important aspect to be considered is represented by the coagulation abnormalities attributed to COVID-19 and their impact on RV function. RV dysfunction is associated with a worse outcome in COVID-19 patients [152]. Moreover, RV dysfunction may appear as a continuous phenomenon in recovered patients with a history of COVID-19-related pulmonary embolism, alveolar and endothelial injury and thrombotic microangiopathy [153]. Up to 42% of recovered COVID-19 patients may present abnormal RV free wall strain, without the presence of other RV structural modifications or in the absence of criteria for pulmonary hypertension, being linked to ongoing inflammation, ischemic lesions due to hypoxia or consequently RV injury post-mechanical ventilation [153]. Yet, it remains difficult to understand their clinical significance in the context of Long COVID.

## 6. Long COVID—Potential Management Directions

Management of Long COVID symptomatology remains a clinical challenge in terms of individual patient care as well as for healthcare and economic institutions. Physical deconditioning and reduced exercise capacity have a crucial impact on patients’ quality of life and long-term prognosis [52]. Rehabilitation centered on individualized exercise training programs may be the first step in improving cardiovascular fitness and diminishing the effects of Long COVID. After 6 weeks of physical and respiratory rehabilitation programs, an amelioration was observed in pulmonary functional tests, quality of life and 6 min walking distance test in COVID-19 survivors [154].

Some studies reported the beneficial effects of vitamin C and L-arginine on walking capacity, muscle strength, endothelial function and fatigue in adults with Long COVID [155,156]. Considering their effects on aerobic and anaerobic performance in patients with cardiopulmonary diseases including HF or heart transplant, their potential efficiency in combination with a physical activity rehabilitation program may be one of the key medical strategies for Long COVID patients. A 12-week inspiratory muscle training program performed at home showed a substantial improvement in patients’ wellbeing and exercise tolerance with an amelioration in peak VO2 [157,158]. Other potential therapies have been evaluated in post-acute COVID-19. Molecular hydrogen (H2) is known for its antioxidative, anti-inflammatory, anti-apoptosis and anti-fatigue properties; therefore, a regimen of 14 days of therapy was proposed in a small cohort of patients, showing positive effects on patients’ physical capacity and wellbeing [159]. Individualized patient treatment and precise diagnosis steps are needed in order to address this new condition. In cases of pulmonary fibrosis following acute COVID-19, targeted therapies including antifibrotic agents are necessary. Several studies focused on the effect of pirfenidone or nintedanib in patients with fibrosis following COVID-19 pneumonia, showing significant amelioration in pulmonary functional tests, oxygen saturation and heart rate, as well as less radiological abnormalities [160,161]. In patients with persistent smell or taste disorders following COVID-19 infection combined treatment, olfactory rehabilitation with oral supplementation of palmitoylethanolamide and luteolin showed beneficial effects on olfactory function recovery [162]. Therefore, due to new emerging therapies in combination with different algorithms designed for rehabilitation programs, there is a potential place for developing Long COVID clinics to address the current issue. The heterogeneity of the Long COVID spectrum needs to be answered in a controlled medical environment, focused on patients’ needs and expectations. Several reports emphasized specific features in order to design Long COVID multidisciplinary clinics capable of addressing the current gaps in healthcare management and reducing the socio-economic factors that may restrain patients from seeking healthcare support [163,164]. Those reports showed the advantage of designing COVID-19 structures centered on rehabilitation programs, including respiratory and cardiology evaluations in addition to neurological and psychological support depending on patients’ main requirements [163,164]. In addition to a faster recovery after COVID-19, those healthcare structures may in time become independent healthcare services, proving management and financial autonomy, by referring patients to other specialized healthcare sectors and by reducing the current burden of Long COVID on the healthcare sector. Importantly, this may promote future research for individualized patient treatment programs after severe infectious diseases including COVID-19.

## 7. Controversial and Unresolved Issues in COVID-19

Despite a growing body of evidence showing the multisystemic effect of COVID-19 pneumonia, data on cardiovascular involvement and its long-term evolution remain insufficiently understood. It is reasonable to conclude that the management of COVID-19 survivors should be guided based on their core needs and specific profiles, including the evolution of previous comorbidities, vaccination status or potential secondary effects caused by COVID-19 treatment during the acute phase. Moreover, the high spectrum of symptoms reported by patients remains difficult to alleviate in current clinical settings due to diagnostic challenges with potential other diseases and the absence of diagnostic and management algorithms for Long COVID.

There is no certainty that post-acute COVID-19 symptoms will fully disappear or if they will have a long-term impact on the quality of life and, furthermore, on the life expectancy of COVID-19 survivors. Hence, further research is needed in this field to answer all these questions; however, it remains a challenge due to a high loss to follow-up rates and unvalidated and discordant protocol methodologies.

## 8. Conclusions and Future Perspectives

The importance of early recognition of Long COVID as a distinct clinical entity is crucial in order to develop specific programs for COVID-19 survivors to reduce its burden on quality of life and on healthcare sectors worldwide. Although new evidence on COVID-19 cardiovascular impact has emerged, we are still too early in the evolution of the COVID-19 pandemic to understand its long-term impact. Therefore, active screening for potential new onset of cardiovascular diseases in this specific population is essential in order to implement preventive cardiovascular measurements. Long COVID clinics appear to have a major role in identifying patients at risk of long-term sequelae, including respiratory and cardiovascular complications, in order to design individualized medical management encompassing cardiopulmonary rehabilitation programs and new Long COVID targeted therapies. Therefore, promoting research programs focused on better understanding the heterogeneity of the pathophysiological mechanisms behind Long COVID, such as direct viral action, chronic inflammation or autoimmunity, in addition to developing new treatment algorithms, is mandatory for improving patients’ prognosis in terms of quality of life and survivorship.

## Data Availability

Not applicable.

## References

[1] Davis H.E., McCorkell L., Vogel J.M., Topol E.J. (2023). Long COVID: Major findings, mechanisms and recommendations. Nat. Rev. Microbiol..

[2] Nalbandian A., Sehgal K., Gupta A., Madhavan M.V., McGroder C., Stevens J.S., Cook J.R., Nordvig A.S., Shalev D., Sehrawat T.S. (2023). Post-acute COVID-19 syndrome. Nat. Med..

[3] Soriano J.B., Murthy S., Marshall J.C., Relan P., Diaz J.V. (2022). A clinical case definition of post-COVID-19 condition by a Delphi consensus. Lancet Infect. Dis..

[4] Davis H.E., Assaf G.S., McCorkell L., Wei H., Low R.J., Re’em Y., Redfield S., Austin J.P., Akrami A. (2021). Characterizing long COVID in an international cohort: 7 months of symptoms and their impact. EClinicalMedicine.

[5] Cazé A.B., Cerqueira-Silva T., Bomfim A.P., de Souza G.L., Azevedo A.C.A., Brasil M.Q.A., Santos N.R., Khouri R., Dan J., Bandeira A.C. (2023). Prevalence and risk factors for long COVID after mild disease: A cohort study with a symptomatic control group. J. Glob. Health.

[6] Swank Z., Senussi Y., Manickas-Hill Z., Yu X.G., Li J.Z., Alter G., Walt D.R. (2023). Persistent Circulating Severe Acute Respiratory Syndrome Coronavirus 2 Spike Is Associated with Post-acute Coronavirus Disease 2019 Sequelae. Clin. Infect. Dis..

[7] Chertow D., Stein S., Ramelli S., Grazioli A., Singh M., Yinda C.K., Winkler C., Dickey J., Ylaya K. SARS-CoV-2 Infection and Persistence Throughout the Human Body and Brain. https://www.researchsquare.com.

[8] Hernández-Parra H., Reyes-Hernández O.D., Figueroa-González G., González-Del Carmen M., González-Torres M., Peña-Corona S.I., Florán B., Cortés H., Leyva-Gómez G. (2023). Alteration of the blood-brain barrier by COVID-19 and its implication in the permeation of drugs into the brain. Front. Cell. Neurosci..

[9] Perico L., Morigi M., Galbusera M., Pezzotta A., Gastoldi S., Imberti B., Perna A., Ruggenenti P., Donadelli R., Benigni A. (2022). SARS-CoV-2 Spike Protein 1 Activates Microvascular Endothelial Cells and Complement System Leading to Platelet Aggregation. Front. Immunol..

[10] Maamar M., Artime A., Pariente E., Fierro P., Ruiz Y., Gutiérrez S., Tobalina M., Díaz-Salazar S., Ramos C., Olmos J.M. (2022). Post-COVID-19 syndrome, low-grade inflammation and inflammatory markers: A cross-sectional study. Curr. Med. Res. Opin..

[11] Antonelli M., Pujol J.C., Spector T.D., Ourselin S., Steves C.J. (2022). Risk of long COVID associated with delta versus omicron variants of SARS-CoV-2. Lancet.

[12] Fernández-de-las-Peñas C., Notarte K.I., Peligro P.J., Velasco J.V., Ocampo M.J., Henry B.M., Arendt-Nielsen L., Torres-Macho J., Plaza-Manzano G. (2022). Long-COVID Symptoms in Individuals Infected with Different SARS-CoV-2 Variants of Concern: A Systematic Review of the Literature. Viruses.

[13] Azzolini E., Levi R., Sarti R., Pozzi C., Mollura M., Mantovani A., Rescigno M. (2022). Association Between BNT162b2 Vaccination and Long COVID After Infections Not Requiring Hospitalization in Health Care Workers. JAMA.

[14] Ayoubkhani D., Bermingham C., Pouwels K.B., Glickman M., Nafilyan V., Zaccardi F., Khunti K., A Alwan N., Walker A.S. (2022). Trajectory of long covid symptoms after COVID-19 vaccination: Community based cohort study. BMJ.

[15] Watanabe A., Iwagami M., Yasuhara J., Takagi H., Kuno T. (2023). Protective effect of COVID-19 vaccination against long COVID syndrome: A systematic review and meta-analysis. Vaccine.

[16] Nicolai L., Kaiser R., Stark K. (2023). Thromboinflammation in long COVID—The elusive key to postinfection sequelae?. J. Thromb. Haemost..

[17] Brodin P. (2021). Immune determinants of COVID-19 disease presentation and severity. Nat Med..

[18] Wollborn J., Karamnov S., Fields K.G., Yeh T., Muehlschlegel J.D. (2022). COVID-19 increases the risk for the onset of atrial fibrillation in hospitalized patients. Sci. Rep..

[19] Bégin P., Callum J., Jamula E., Cook R., Heddle N.M., Tinmouth A., Zeller M.P., Amorim L., Loftsgard K.C., Carl R. (2021). Convalescent plasma for hospitalized patients with COVID-19: An open-label, randomized controlled trial. Nat. Med..

[20] Price L.C., McCabe C., Garfield B., Wort S.J. (2020). Thrombosis and COVID-19 pneumonia: The clot thickens!. Eur. Respir. J..

[21] Nishiga M., Wang D.W., Han Y., Lewis D.B., Wu J.C. (2020). COVID-19 and cardiovascular disease: From basic mechanisms to clinical perspectives. Nat. Rev. Cardiol..

[22] Toraih E.A., Elshazli R.M., Hussein M.H., Elgaml A., Amin M., El-Mowafy M., El-Mesery M., Ellythy A., Duchesne J., Killackey M.T. (2020). Association of cardiac biomarkers and comorbidities with increased mortality, severity, and cardiac injury in COVID-19 patients: A meta-regression and decision tree analysis. J. Med. Virol..

[23] Lodigiani C., Iapichino G., Carenzo L., Cecconi M., Ferrazzi P., Sebastian T., Kucher N., Studt J.D., Sacco C., Bertuzzi A. (2020). Venous and arterial thromboembolic complications in COVID-19 patients admitted to an academic hospital in Milan, Italy. Thromb. Res..

[24] Fogarty H., Townsend L., Morrin H., Ahmad A., Comerford C., Karampini E., Englert H., Byrne M., Bergin C., O’Sullivan J.M. (2021). Persistent endotheliopathy in the pathogenesis of long COVID syndrome. J. Thromb. Haemost..

[25] Su Y., Yuan D., Chen D.G., Ng R.H., Wang K., Choi J., Li S., Hong S., Zhang R., Xie J. (2022). Multiple early factors anticipate post-acute COVID-19 sequelae. Cell.

[26] Forshaw D., Wall E.C., Prescott G., Dehbi H.M., Green A., Attree E., Hismeh L., Strain W.D., Crooks M.G., Watkins C. (2023). STIMULATE-ICP: A pragmatic, multi-centre, cluster randomised trial of an integrated care pathway with a nested, Phase III, open label, adaptive platform randomised drug trial in individuals with Long COVID: A structured protocol. PLoS ONE.

[27] A Decentralized, Randomized Phase 2 Efficacy and Safety Study of Nirmatrelvir/Ritonavir in Adults with Long COVID—Full Text View—ClinicalTrials.gov. https://classic.clinicaltrials.gov/ct2/show/NCT05668091?cond=Long+COVID&draw=2&rank=2.

[28] Peluso M.J., Deveau T.M., Munter S.E., Ryder D., Buck A., Beck-Engeser G., Chan F., Lu S., Goldberg S.A., Hoh R. (2023). Chronic viral coinfections differentially affect the likelihood of developing long COVID. J. Clin. Investig..

[29] Tsilingiris D., Vallianou N.G., Karampela I., Christodoulatos G.S., Papavasileiou G., Petropoulou D., Magkos F., Dalamaga M. (2023). Laboratory Findings and Biomarkers in Long COVID: What Do We Know So Far? Insights into Epidemiology, Pathogenesis, Therapeutic Perspectives and Challenges. Int. J. Mol. Sci..

[30] Leppkes M., Neurath M.F. (2022). Rear Window—What Can the Gut Tell Us About Long-COVID?. Gastroenterology.

[31] Zollner A., Koch R., Jukic A., Pfister A., Meyer M., Rössler A., Kimpel J., Adolph T.E., Tilg H. (2022). Postacute COVID-19 is Characterized by Gut Viral Antigen Persistence in Inflammatory Bowel Diseases. Gastroenterology.

[32] Phetsouphanh C., Darley D.R., Wilson D.B., Howe A., Munier C.M.L., Patel S.K., Juno J.A., Burrell L.M., Kent S.J., Dore G.J. (2022). Immunological dysfunction persists for 8 months following initial mild-to-moderate SARS-CoV-2 infection. Nat. Immunol..

[33] Zhou Y., Zhang J., Zhang D., Ma W.L., Wang X. (2021). Linking the gut microbiota to persistent symptoms in survivors of COVID-19 after discharge. J. Microbiol..

[34] Zhou Y., Shi X., Fu W., Xiang F., He X., Yang B., Wang X., Ma W.L. (2021). Gut Microbiota Dysbiosis Correlates with Abnormal Immune Response in Moderate COVID-19 Patients with Fever. J. Inflamm. Res..

[35] Liu Q., Mak J.W.Y., Su Q., Yeoh Y.K., Lui G.C.Y., Ng S.S.S., Zhang F., Li A.Y.L., Lu W., Hui D.S.-C. (2022). Gut microbiota dynamics in a prospective cohort of patients with post-acute COVID-19 syndrome. Gut.

[36] Zuo T., Wu X., Wen W., Lan P. (2021). Gut Microbiome Alterations in COVID-19. Genom. Proteom. Bioinform..

[37] Lai C.C., Hsu C.K., Yen M.Y., Lee P.I., Ko W.C., Hsueh P.R. (2023). Long COVID: An inevitable sequela of SARS-CoV-2 infection. J. Microbiol. Immunol. Infect..

[38] Al-Aly Z., Bowe B., Xie Y. (2022). Long COVID after breakthrough SARS-CoV-2 infection. Nat. Med..

[39] Saloner B., Parish K., Ward J.A., Dilaura G., Dolovich S. (2020). COVID-19 cases and deaths in federal and state prisons. JAMA J. Am. Med. Assoc..

[40] Saloner B., Parish K., Julie Ward M.A., Grace DiLaura R., Sharon Dolovich J. (2020). Persistent Symptoms in Patients After Acute COVID-19. JAMA.

[41] Zawilska J.B., Kuczyńska K. (2022). Psychiatric and neurological complications of long COVID. J Psychiatr Res..

[42] Alkodaymi M.S., Omrani O.A., Fawzy N.A., Shaar B.A., Almamlouk R., Riaz M., Obeidat M., Obeidat Y., Gerberi D., Taha R.M. (2022). Prevalence of post-acute COVID-19 syndrome symptoms at different follow-up periods: A systematic review and meta-analysis. Clin. Microbiol. Infect..

[43] Sykes D.L., Holdsworth L., Jawad N., Gunasekera P., Morice A.H., Crooks M.G. (2021). Post-COVID-19 Symptom Burden: What is Long-COVID and How Should We Manage It?. Lung.

[44] Eggers K.M., Jernberg T., Lindahl B. (2019). Cardiac Troponin Elevation in Patients Without a Specific Diagnosis. J. Am. Coll. Cardiol..

[45] Linschoten M., Peters S., van Smeden M., Jewbali L.S., Schaap J., Siebelink H.M., Smits P.C., Tieleman R.G., van der Harst P., van Gilst W.H. (2020). Cardiac complications in patients hospitalised with COVID-19. Eur. Heart J. Acute Cardiovasc. Care.

[46] Chapman A.R., Bularga A., Mills N.L. (2020). High-Sensitivity Cardiac Troponin Can Be an Ally in the Fight Against COVID-19. Circulation.

[47] Luchian M.L., Motoc A.I., Lochy S., Magne J., Roosens B., Belsack D., Van den Bussche K., von Kemp B., Galloo X., François C. (2021). Troponin T in COVID-19 hospitalized patients: Kinetics matter. Cardiol. J..

[48] Giustino G., Croft L.B., Stefanini G.G., Bragato R., Silbiger J.J., Vicenzi M., Danilov T., Kukar N., Shaban N., Kini A. (2020). Characterization of Myocardial Injury in Patients With COVID-19. J. Am. Coll. Cardiol..

[49] Huang L., Zhao P., Tang D., Zhu T., Han R., Zhan C., Liu W., Zeng H., Tao Q., Xia L. (2020). Cardiac Involvement in Patients Recovered From COVID-2019 Identified Using Magnetic Resonance Imaging. JACC Cardiovasc. Imaging.

[50] Puntmann V.O., Carerj M.L., Wieters I., Fahim M., Arendt C., Hoffmann J., Shchendrygina A., Escher F., Vasa-Nicotera M., Zeiher A.M. (2020). Outcomes of Cardiovascular Magnetic Resonance Imaging in Patients Recently Recovered from Coronavirus Disease 2019 (COVID-19). JAMA Cardiol..

[51] Xu H., Hou K., Xu R., Li Z., Fu H., Wen L., Xie L., Liu H., Selvanayagam J.B., Zhang N. (2020). Clinical Characteristics and Risk Factors of Cardiac Involvement in COVID-19. J. Am. Heart Assoc..

[52] Sonnweber T., Sahanic S., Pizzini A., Luger A., Schwabl C., Sonnweber B., Kurz K., Koppelstätter S., Haschka D., Petzer V. (2021). Cardiopulmonary recovery after COVID-19: An observational prospective multicentre trial. Eur. Respir. J..

[53] Minhas A.S., Gilotra N.A., Goerlich E., Metkus T., Garibaldi B.T., Sharma G., Bavaro N., Phillip S., Michos E.D., Hays A.G. (2021). Myocardial Work Efficiency, A Novel Measure of Myocardial Dysfunction, Is Reduced in COVID-19 Patients and Associated with In-Hospital Mortality. Front. Cardiovasc. Med..

[54] Kotecha T., Knight D.S., Razvi Y., Kumar K., Vimalesvaran K., Thornton G., Patel R., Chacko L., Brown J.T., Coyle C. (2021). Patterns of myocardial injury in recovered troponin-positive COVID-19 patients assessed by cardiovascular magnetic resonance. Eur. Heart J..

[55] Diaz-Arocutipa C., Saucedo-Chinchay J., Imazio M. (2021). Pericarditis in patients with COVID-19: A systematic review. J. Cardiovasc. Med..

[56] Tam C.-C.F., Siu D., Tse H.F. (2022). COVID-19 and Acute Coronary Syndrome: Lessons for Everyone. Lancet Reg. Health West Pac..

[57] Kite T.A., Ludman P.F., Gale C.P., Wu J., Caixeta A., Mansourati J., Sabate M., Jimenez-Quevedo P., Candilio L., Sadeghipour P. (2021). International Prospective Registry of Acute Coronary Syndromes in Patients with COVID-19. J. Am. Coll. Cardiol..

[58] Esposito L., Cancro F.P., Silverio A., Di Maio M., Iannece P., Damato A., Alfano C., De Luca G., Vecchione C., Galasso G. (2021). COVID-19 and Acute Coronary Syndromes: From Pathophysiology to Clinical Perspectives. Oxid Med. Cell. Longev..

[59] Shah R.M., Shah M., Shah S., Li A., Jauhar S. (2021). Takotsubo Syndrome and COVID-19: Associations and Implications. Curr. Probl. Cardiol..

[60] Vidula M.K., Rajewska-Tabor J., Cao J.J., Kang Y., Craft J., Mei W., Chandrasekaran P.S., Clark D.E., Poenar A.-M., Gorecka M. (2023). Myocardial Injury on CMR in Patients with COVID-19 and Suspected Cardiac Involvement. JACC Cardiovasc. Imaging.

[61] Ammirati E., Lupi L., Palazzini M., Hendren N.S., Grodin J.L., Cannistraci C.V., Schmidt M., Hekimian G., Peretto G., Bochaton T. (2022). Prevalence, Characteristics, and Outcomes of COVID-19–Associated Acute Myocarditis. Circulation.

[62] Rath D., Petersen-Uribe Á., Avdiu A., Witzel K., Jaeger P., Zdanyte M., Heinzmann D., Tavlaki E., Müller K., Gawaz M.P. (2020). Impaired cardiac function is associated with mortality in patients with acute COVID-19 infection. Clin. Res. Cardiol..

[63] Petersen S.E., Friedrich M.G., Leiner T., Elias M.D., Ferreira V.M., Fenski M., Flamm S.D., Fogel M., Garg R., Halushka M.K. (2022). Cardiovascular Magnetic Resonance for Patients With COVID-19. JACC Cardiovasc. Imaging.

[64] Siripanthong B., Asatryan B., Hanff T.C., Chatha S.R., Khanji M.Y., Ricci F., Muser D., Ferrari V.A., Nazarian S., Santangeli P. (2022). The Pathogenesis and Long-Term Consequences of COVID-19 Cardiac Injury. JACC Basic Transl. Sci..

[65] Gorecka M., Jex N., Thirunavukarasu S., Chowdhary A., Corrado J., Davison J., Tarrant R., Poenar A.-M., Sharrack N., Parkin A. (2022). Cardiovascular magnetic resonance imaging and spectroscopy in clinical long-COVID-19 syndrome: A prospective case–control study. J. Cardiovasc. Magn. Reson..

[66] Joy G., Artico J., Kurdi H., Seraphim A., Lau C., Thornton G.D., Oliveira M.F., Adam R.D., Aziminia N., Menacho K. (2021). Prospective Case-Control Study of Cardiovascular Abnormalities 6 Months Following Mild COVID-19 in Healthcare Workers. JACC Cardiovasc. Imaging.

[67] Puntmann V.O., Martin S., Shchendrygina A., Hoffmann J., Ka M.M., Giokoglu E., Vanchin B., Holm N., Karyou A., Laux G.S. (2022). Long-term cardiac pathology in individuals with mild initial COVID-19 illness. Nat. Med..

[68] Wojtowicz D., Dorniak K., Ławrynowicz M., Wąż P., Fijałkowska J., Kulawiak-Gałąska D., Rejszel-Baranowska J., Knut R., Haberka M., Szurowska E. (2022). Cardiac Magnetic Resonance Findings in Patients Recovered from COVID-19 Pneumonia and Presenting with Persistent Cardiac Symptoms: The TRICITY-CMR Trial. Biology.

[69] Yar A., Uusitalo V., Vaara S.M., Holmström M., Vuorinen A.M., Heliö T., Paakkanen R., Kivistö S., Syväranta S., Hästbacka J. (2023). Cardiac magnetic resonance -detected myocardial injury is not associated with long-term symptoms in patients hospitalized due to COVID-19. PLoS ONE.

[70] Gupta M., Kunal S., Bagarhatta P., Girish M.P., Bansal A., Batra V., Daga M., Tyagi S., Sharma A., Bansal K. (2022). Utility of cardiovascular magnetic resonance imaging in COVID-19 recovered patients: A short-term follow-up study. Authorea Preprints.

[71] Fernandez A.R., Wamil M., Telford A., Carapella V., Borlotti A., Monteiro D., Thomaides-Brears H., Kelly M., Dennis A., Banerjee R. (2023). Original research: Cardiac abnormalities in Long COVID 1-year post-SARS-CoV-2 infection. Open Heart.

[72] Luchian M.L., Motoc A., Lochy S., Magne J., Belsack D., De Mey J., Roosens B., Van den Bussche K., Boeckstaens S., Chameleva H. (2022). Subclinical Myocardial Dysfunction in Patients with Persistent Dyspnea One Year after COVID-19. Diagnostics.

[73] Ikonomidis I., Lambadiari V., Mitrakou A., Kountouri A., Katogiannis K., Thymis J., Korakas E., Pavlidis G., Kazakou P., Panagopoulos G. (2022). Myocardial work and vascular dysfunction are partially improved at 12 months after COVID-19 infection. Eur. J. Heart Fail..

[74] Elseidy S.A., Awad A.K., Vorla M., Fatima A., Elbadawy M.A., Mandal D., Mohamad T. (2022). Cardiovascular complications in the Post-Acute COVID-19 syndrome (PACS). IJC Heart Vasc..

[75] Shi S., Qin M., Shen B., Cai Y., Liu T., Yang F., Gong W., Liu X., Liang J., Zhao Q. (2020). Association of Cardiac Injury with Mortality in Hospitalized Patients with COVID-19 in Wuhan, China. JAMA Cardiol..

[76] Fu L., Liu X., Su Y., Ma J., Hong K. (2021). Prevalence and impact of cardiac injury on COVID-19: A systematic review and meta-analysis. Clin. Cardiol..

[77] Sandoval Y., Januzzi J.L., Jaffe A.S. (2020). Cardiac Troponin for Assessment of Myocardial Injury in COVID-19: JACC Review Topic of the Week. J. Am. Coll. Cardiol..

[78] Luan Y.Y., Yin C.H., Yao Y.M. Update Advances on C-Reactive Protein in COVID-19 and Other Viral Infections 2019. www.frontiersin.org.

[79] Otifi H.M., Adiga B.K. (2022). Endothelial Dysfunction in COVID-19 Infection. Am. J. Med. Sci..

[80] Jin Y., Ji W., Yang H., Chen S., Zhang W., Duan G. (2020). Endothelial activation and dysfunction in COVID-19: From basic mechanisms to potential therapeutic approaches. Signal Transduct. Target. Ther..

[81] Fagyas M., Nagy B., Ráduly A.P., Mányiné I.S., Mártha L., Erdősi G., Sipka S., Enyedi E., Szabó A., Pólik Z. (2022). The majority of severe COVID-19 patients develop anti-cardiac autoantibodies. Geroscience.

[82] Stelzer M., Henes J., Saur S. (2021). The Role of Antiphospholipid Antibodies in COVID-19. Curr. Rheumatol. Rep..

[83] Xie Y., Xu E., Bowe B., Al-Aly Z. (2022). Long-term cardiovascular outcomes of COVID-19. Nat. Med..

[84] Bowe B., Xie Y., Al-Aly Z. (2023). Postacute sequelae of COVID-19 at 2 years. Nat. Med..

[85] Bernard C., Andreea M., Luiza L.M., Stijn L., Dries B. (2020). Coronary Calcium Score in COVID-19 Hospitalized Patients. JACC Cardiovasc. Imaging.

[86] Cosyns B., Lochy S., Luchian M.L., Gimelli A., Pontone G., Allard S.D., De Mey J., Rosseel P., Dweck M., E Petersen S. (2020). The role of cardiovascular imaging for myocardial injury in hospitalized COVID-19 patients. Eur. Heart J. Cardiovasc. Imaging.

[87] Dani M., Dirksen A., Taraborrelli P., Torocastro M., Panagopoulos D., Sutton R., Lim P.B. (2021). Autonomic dysfunction in ‘long COVID’: Rationale, physiology and management strategies. Clin. Med..

[88] Freeman R., Abuzinadah A.R., Gibbons C., Jones P., Miglis M.G., Sinn D.I. (2018). Orthostatic Hypotension: JACC State-of-the-Art Review. J. Am. Coll. Cardiol..

[89] Bryarly M., Phillips L.T., Fu Q., Vernino S., Levine B.D. (2019). Postural Orthostatic Tachycardia Syndrome: JACC Focus Seminar. J. Am. Coll. Cardiol..

[90] Spahic J.M., Mattisson I.Y., Hamrefors V., Johansson M., Ricci F., Nilsson J., Melander O., Sutton R., Fedorowski A. (2023). Evidence for Impaired Renin Activity in Postural Orthostatic Tachycardia Syndrome. J. Clin. Med..

[91] Rysz S., Al-Saadi J., Sjöström A., Farm M., Campoccia Jalde F., Plattén M., Eriksson H., Klein M., Vargas-Paris R., Nyrén S. (2021). COVID-19 pathophysiology may be driven by an imbalance in the renin-angiotensin-aldosterone system. Nat. Commun..

[92] Mustafa H.I., Raj S.R., Diedrich A., Black B.K., Paranjape S.Y., Dupont W.D., Williams G.H., Biaggioni I., Robertson D. (2012). Altered systemic hemodynamic and baroreflex response to angiotensin II in postural tachycardia syndrome. Circ. Arrhythm. Electrophysiol..

[93] Mar P.L., Raj S.R. (2014). Neuronal and hormonal perturbations in postural tachycardia syndrome. Front. Physiol..

[94] Yesudhas D., Srivastava A., Gromiha M.M. (2020). COVID-19 outbreak: History, mechanism, transmission, structural studies and therapeutics. Infection.

[95] Chen L., Li X., Chen M., Feng Y., Xiong C. (2020). The ACE2 expression in human heart indicates new potential mechanism of heart injury among patients infected with SARS-CoV-2. Cardiovasc. Res..

[96] Oudit G.Y., Kassiri Z., Jiang C., Liu P.P., Poutanen S.M., Penninger J.M., Butany J. (2009). SARS-coronavirus modulation of myocardial ACE2 expression and inflammation in patients with SARS. Eur. J. Clin. Investig..

[97] Hamming I., Timens W., Bulthuis M.L.C., Lely A.T., Navis G.J., van Goor H. (2004). Tissue distribution of ACE2 protein, the functional receptor for SARS coronavirus. A first step in understanding SARS pathogenesis. J. Pathol..

[98] Kanjwal K., Jamal S., Kichloo A., Grubb B.P. (2020). New-onset Postural Orthostatic Tachycardia Syndrome Following Coronavirus Disease 2019 Infection. J. Innov. Card. Rhythm..

[99] Chadda K.R., Blakey E.E., Huang C.L.H., Jeevaratnam K. (2022). Long COVID-19 and Postural Orthostatic Tachycardia SyndromeIs Dysautonomia to Be Blamed?. Front. Cardiovasc. Med..

[100] Shouman K., Vanichkachorn G., Cheshire W.P., Suarez M.D., Shelly S., Lamotte G.J., Sandroni P., Benarroch E.E., Berini S.E., Cutsforth-Gregory J.K. (2021). Autonomic dysfunction following COVID-19 infection: An early experience. Clin. Auton. Res..

[101] Narasimhan B., Calambur A., Moras E., Wu L., Aronow W. (2023). Postural Orthostatic Tachycardia Syndrome in COVID-19: A Contemporary Review of Mechanisms, Clinical Course and Management. Vasc. Health Risk Manag..

[102] Johansson M., Ståhlberg M., Runold M., Nygren-Bonnier M., Nilsson J., Olshansky B., Bruchfeld J., Fedorowski A. (2021). Long-Haul Post–COVID-19 Symptoms Presenting as a Variant of Postural Orthostatic Tachycardia Syndrome: The Swedish Experience. JACC Case Rep..

[103] Isaac R.O., Corrado J., Sivan M. (2023). Detecting Orthostatic Intolerance in Long COVID in a Clinic Setting. Int. J. Environ. Res. Public Health.

[104] Jamal S.M., Landers D.B., Hollenberg S.M., Turi Z.G., Glotzer T.V., Tancredi J., Parrillo J.E. (2022). Prospective Evaluation of Autonomic Dysfunction in Post-Acute Sequela of COVID-19. J Am. Coll. Cardiol..

[105] Van Campen C (Linda) M.C., Visser F.C. (2022). Orthostatic Intolerance in Long-Haul COVID after SARS-CoV-2: A Case-Control Comparison with Post-EBV and Insidious-Onset Myalgic Encephalomyelitis/Chronic Fatigue Syndrome Patients. Healthcare.

[106] Monaghan A., Jennings G., Xue F., Byrne L., Duggan E., Romero-Ortuno R. (2022). Orthostatic Intolerance in Adults Reporting Long COVID Symptoms Was Not Associated with Postural Orthostatic Tachycardia Syndrome. Front. Physiol..

[107] Ormiston C.K., Świątkiewicz I., Taub P.R. (2022). Postural orthostatic tachycardia syndrome as a sequela of COVID-19. Heart Rhythm..

[108] ZeinElabdeen S.G., Sherif A., Kandil N.T., Altabib A.M.O., Abdelrashid M.A. (2023). Left atrial longitudinal strain analysis in long COVID-19 syndrome. Int. J. Cardiovasc. Imaging.

[109] Hopman L.H.G.A., Mulder M.J., van der Laan A.M., Demirkiran A., Bhagirath P., van Rossum A.C., Allaart C.P., Götte M.J. (2021). Impaired left atrial reservoir and conduit strain in patients with atrial fibrillation and extensive left atrial fibrosis. J. Cardiovasc. Magn. Reason..

[110] Motoc A., Luchian M.L., Scheirlynck E., Roosens B., Chameleva H., Gevers M., Galloo X., von Kemp B., Ramak R., Sieira J. (2021). Incremental value of left atrial strain to predict atrial fibrillation recurrence after cryoballoon ablation. PLoS ONE.

[111] Goerlich E., Minhas A., Gilotra N., Barth A.S., Mukherjee M., Parziale A., Wu K.C., Hays A.G. (2021). Left Atrial Function in Patients with Coronavirus Disease 2019 and Its Association with Incident Atrial Fibrillation/Flutter. J. Am. Soc. Echocardiogr..

[112] Beyls C., Hermida A., Bohbot Y., Martin N., Viart C., Boisgard S., Daumin C., Huette P., Dupont H., Abou-Arab O. (2021). Automated left atrial strain analysis for predicting atrial fibrillation in severe COVID-19 pneumonia: A prospective study. Ann. Intensive Care.

[113] Huseynov A., Akin I., Duerschmied D., Scharf R.E. (2023). Cardiac Arrhythmias in Post-COVID Syndrome: Prevalence, Pathology, Diagnosis, and Treatment. Viruses.

[114] Donniacuo M., De Angelis A., Rafaniello C., Cianflone E., Paolisso P., Torella D., Sibilio G., Paolisso G., Castaldo G., Urbanek K. (2023). COVID-19 and atrial fibrillation: Intercepting lines. Front. Cardiovasc. Med..

[115] Tarantino N., Della Rocca D.G., Zou F., Lin A., Natale A., Di Biase L. (2022). Prevalence, Outcomes, and Management of Ventricular Arrhythmias in COVID-19 Patients. Card. Electrophysiol. Clin..

[116] Long B., Brady W.J., Bridwell R.E., Ramzy M., Montrief T., Singh M., Gottlieb M. (2021). Electrocardiographic manifestations of COVID-19. Am. J. Emerg. Med..

[117] Nagamine T., Randhawa S., Nishimura Y., Huang R., Leesutipornchai T., Benavente K., Yoshimura S., Zhang J., Kanitsorphan C. (2022). Characteristics of bradyarrhythmia in patients with COVID-19: Systematic scoping review. Pacing Clin. Electrophysiol..

[118] Etheridge S.P., Asaki S.Y. (2021). COVID-19 Infection and Corrected QT Interval Prolongation—Collateral Damage from Our Newest Enemy. JAMA Netw. Open.

[119] Charlton F.W., Pearson H.M., Hover S., Lippiat J.D., Fontana J., Barr J.N., Mankouri J. (2020). Ion Channels as Therapeutic Targets for Viral Infections: Further Discoveries and Future Perspectives. Viruses.

[120] Carlson F.R., Bosukonda D., Keck P.C., Carlson W.D. (2020). Multiorgan Damage in Patients With COVID-19: Is the TGF-β/BMP Pathway the Missing Link?. JACC Basic Transl. Sci..

[121] Wang E.Y., Chen H., Sun B.Q., Wang H., Qu H.Q., Liu Y., Sun X., Qu J., Fang Z., Tian L. (2021). Serum levels of the IgA isotype switch factor TGF-β1 are elevated in patients with COVID-19. FEBS Lett..

[122] Mormile R. (2022). High degree atrioventricular block and COVID-19 infection: A two player match?. Expert Rev. Cardiovasc. Ther..

[123] Garcia-Zamora S., Lee S., Haseeb S., Bazoukis G., Tse G., Alvarez-Garcia J., Gul E.E., Çinier G., Alexander B., Pinto-Filho M.M. (2021). Arrhythmias and electrocardiographic findings in Coronavirus disease 2019: A systematic review and meta-analysis. Pacing Clin. Electrophysiol..

[124] Liliequist D.A., Svensson P.P., Hoffmann A.P.R., Habel D.H., Nordberg A.P.P., Stahlberg D.M. (2023). Surviving critical COVID-19 requiring mechanical ventilation is associated with a high long-term risk of de novo arrhythmic events. Europace.

[125] Wang W., Wang C.Y., Wang S.I., Wei J.C.C. (2022). Long-term cardiovascular outcomes in COVID-19 survivors among non-vaccinated population: A retrospective cohort study from the TriNetX US collaborative networks. EClinicalMedicine.

[126] Raman B., Bluemke D.A., Lüscher T.F., Neubauer S. (2022). Long COVID: Post-acute sequelae of COVID-19 with a cardiovascular focus. Eur. Heart J..

[127] Collaborative T.O., Tazare J., Walker A.J., Tomlinson L., Hickman G., Rentsch C.T., Williamson E.J., Bhaskaran K., Evans D., Wing K. (2021). Rates of serious clinical outcomes in survivors of hospitalisation with COVID-19: A descriptive cohort study within the OpenSAFELY platform. medRxiv.

[128] Jung L.Y., Kim J.M., Ryu S., Lee C.S. (2022). Relative bradycardia in patients with COVID-19. Int. J. Arrhythmia.

[129] Ye F., Winchester D., Stalvey C., Jansen M., Lee A., Khuddus M., Mazza J., Yale S. (2018). Proposed mechanisms of relative bradycardia. Med. Hypotheses.

[130] Basso C., Leone O., Rizzo S., De Gaspari M., van der Wal A.C., Aubry M.C., Bois M.C., Lin P.T., Maleszewski J.J., Stone J.R. (2020). Pathological features of COVID-19-associated myocardial injury: A multicentre cardiovascular pathology study. Eur. Heart J..

[131] Babapoor-Farrokhran S., Gill D., Walker J., Rasekhi R.T., Bozorgnia B., Amanullah A. (2020). Myocardial injury and COVID-19: Possible mechanisms. Life Sci..

[132] Domanski M.J., Wu C.O., Tian X., Hasan A.A., Ma X., Huang Y., Miao R., Reis J.P., Bae S., Husain A. (2023). Association of Incident Cardiovascular Disease with Time Course and Cumulative Exposure to Multiple Risk Factors. J. Am. Coll. Cardiol..

[133] Standl E., Schnell O. (2021). Heart failure outcomes and COVID-19. Diabetes Res. Clin. Pract..

[134] Ta Anyu A., Badawy L., Cannata A., Bromage D.I., Rind I.A., Albarjas M., Piper S., Shah A.M., McDonagh T.A. (2021). Long-term outcomes after heart failure hospitalization during the COVID-19 pandemic: A multisite report from heart failure referral centers in London. ESC Heart Fail..

[135] Andersson C., Gerds T., Fosbøl E., Phelps M., Andersen J., Lamberts M., Holt A., Butt J.H., Madelaire C., Gislason G. (2020). Incidence of New-Onset and Worsening Heart Failure before and after the COVID-19 Epidemic Lockdown in Denmark: A Nationwide Cohort Study. Circ. Heart Fail..

[136] Zuin M., Rigatelli G., Roncon L., Pasquetto G., Bilato C. (2023). Risk of incident heart failure after COVID-19 recovery: A systematic review and meta-analysis. Heart Fail Rev..

[137] Libby P., Lüscher T. (2020). COVID-19 is, in the end, an endothelial disease. Eur. Heart J..

[138] Gavriilaki E., Eftychidis I., Papassotiriou I. (2021). Update on endothelial dysfunction in COVID-19: Severe disease, long COVID-19 and pediatric characteristics. J. Lab. Med..

[139] Moody W.E., Mahmoud-Elsayed H.M., Senior J., Gul U., Khan-Kheil A.M., Horne S., Banerjee A., Bradlow W.M., Huggett R., Hothi S.S. (2021). Impact of Right Ventricular Dysfunction on Mortality in Patients Hospitalized with COVID-19, according to Race. CJC Open.

[140] Moody W.E., Liu B., Mahmoud-Elsayed H.M., Senior J., Lalla S.S., Khan-Kheil A.M., Brown S., Saif A., Moss A., Bradlow W.M. (2021). Persisting Adverse Ventricular Remodeling in COVID-19 Survivors: A Longitudinal Echocardiographic Study. J. Am. Soc. Echocardiogr..

[141] Eiros R., Barreiro-Perez M., Martin-Garcia A., Almeida J., Villacorta E., Perez-Pons A., Merchan S., Torres-Valle A., Sánchez Pablo C., González-Calle D. (2020). Pericarditis and myocarditis long after SARS-CoV-2 infection: A cross-sectional descriptive study in health-care workers. medRxiv.

[142] Catena C., Colussi G., Bulfone L., Da Porto A., Tascini C., Sechi L.A. (2021). Echocardiographic Comparison of COVID-19 Patients with or without Prior Biochemical Evidence of Cardiac Injury after Recovery. J. Am. Soc. Echocardiogr..

[143] Daniels C.J., Rajpal S., Greenshields J.T., Rosenthal G.L., Chung E.H., Terrin M., Jeudy J., Mattson S.E., Law I.H., Borchers J. (2021). Prevalence of Clinical and Subclinical Myocarditis in Competitive Athletes with Recent SARS-CoV-2 Infection: Results from the Big Ten COVID-19 Cardiac Registry. JAMA Cardiol..

[144] Bader F., Manla Y., Atallah B., Starling R.C. (2021). Heart failure and COVID-19. Heart Fail. Rev..

[145] Szekely Y., Lichter Y., Taieb P., Banai A., Hochstadt A., Merdler I., Oz A.G., Rothschild E., Baruch G., Peri Y. (2020). Spectrum of Cardiac Manifestations in COVID-19. Circulation.

[146] Martins-Gonçalves R., Hottz E.D., Bozza P.T. (2023). Acute to post-acute COVID-19 thromboinflammation persistence: Mechanisms and potential consequences. Curr. Res. Immunol..

[147] artins-Gonçalves R., Campos M.M., Palhinha L., Azevedo-Quintanilha I.G., Abud Mendes M., Ramos Temerozo J., Toledo-Mendes J., Rosado-de-Castro P.H., Bozza F.A., Souza Rodrigues R. (2022). Persisting Platelet Activation and Hyperactivity in COVID-19 Survivors. Circ. Res..

[148] Mohamed I., de Broucker V., Duhamel A., Giordano J., Ego A., Fonne N., Chenivesse C., Remy J., Remy-Jardin M. (2023). Pulmonary circulation abnormalities in post-acute COVID-19 syndrome: Dual-energy CT angiographic findings in 79 patients. Eur. Radiol..

[149] Korompoki E., Gavriatopoulou M., Fotiou D., Ntanasis-Stathopoulos I., Dimopoulos M.A., Terpos E. (2022). Late-onset hematological complications post COVID-19: An emerging medical problem for the hematologist. Am. J. Hematol..

[150] Roberts L.N., Whyte M.B., Georgiou L., Giron G., Czuprynska J., Rea C., Vadher B., Patel R.K., Gee E., Arya R. (2020). Postdischarge venous thromboembolism following hospital admission with COVID-19. Blood.

[151] Zuin M., Rigatelli G., Zuliani G., Roncon L. (2021). The risk of thrombosis after acute-COVID-19 infection. QJM Int. J. Med..

[152] Corica B., Maria Marra A., Basili S., Cangemi R., Cittadini A., Proietti M., Romiti G.F. (2021). Prevalence of right ventricular dysfunction and impact on all-cause death in hospitalized patients with COVID-19: A systematic review and meta-analysis. Sci. Rep..

[153] Nuzzi V., Castrichini M., Collini V., Roman-Pognuz E., Di Bella S., Luzzati R., Berlot G., Confalonieri M., Merlo M., Stolfo D. (2021). Impaired Right Ventricular Longitudinal Strain without Pulmonary Hypertension in Patients Who Have Recovered From COVID-19. Circ. Cardiov. Imaging.

[154] Liu K., Zhang W., Yang Y., Zhang J., Li Y., Chen Y. (2020). Respiratory rehabilitation in elderly patients with COVID-19: A randomized controlled study. Complement Ther. Clin. Pract..

[155] Tosato M., Calvani R., Picca A., Ciciarello F., Galluzzo V., Coelho-Júnior H.J., Di Giorgio A., Di Mario C., Gervasoni J., Gremese E. (2022). Effects of l-Arginine Plus Vitamin C Supplementation on Physical Performance, Endothelial Function, and Persistent Fatigue in Adults with Long COVID: A Single-Blind Randomized Controlled Trial. Nutrients.

[156] Calvani R., Gervasoni J., Picca A., Ciciarello F., Galluzzo V., Coelho-Júnior H.J., Di Mario C., Gremese E., Lomuscio S., Paglionico A.M. (2023). Effects of l-Arginine Plus Vitamin C Supplementation on l-Arginine Metabolism in Adults with Long COVID: Secondary Analysis of a Randomized Clinical Trial. Int. J. Mol. Sci..

[157] Palau P., Domínguez E., Gonzalez C., Bondía E., Albiach C., Sastre C., Martínez M.L., Núñez J., López L. (2022). Effect of a home-based inspiratory muscle training programme on functional capacity in postdischarged patients with long COVID: The InsCOVID trial. BMJ Open Respir. Res..

[158] Jimeno-Almazán A., Buendía-Romero Á., Martínez-Cava A., Franco-López F., Sánchez-Alcaraz B.J., Courel-Ibáñez J., Pallarés J.G. (2023). Effects of a concurrent training, respiratory muscle exercise, and self-management recommendations on recovery from post-COVID-19 conditions: The RECOVE trial. J. Appl. Physiol..

[159] Botek M., Krejčí J., Valenta M., McKune A., Sládečková B., Konečný P., Klimešová I., Pastucha D. (2022). Molecular Hydrogen Positively Affects Physical and Respiratory Function in Acute Post-COVID-19 Patients: A New Perspective in Rehabilitation. Int. J. Environ. Res. Public Health.

[160] Kerget B., Çil G., Araz Ö., Alper F., Akgün M. (2023). Comparison of two antifibrotic treatments for lung fibrosis in post-COVID-19 syndrome: A randomized, prospective study. Med. Clin..

[161] Singh P., Behera D., Gupta S., Deep A., Priyadarshini S., Padhan P. (2022). Nintedanib vs pirfenidone in the management of COVID-19 lung fibrosis: A single-centre study. J. R Coll. Physicians. Edinb..

[162] D’Ascanio L., Vitelli F., Cingolani C., Maranzano M., Brenner M.J., Stadio A.D.I. (2021). Randomized clinical trial “olfactory dysfunction after COVID-19: Olfactory rehabilitation therapy vs. intervention treatment with Palmitoylethanolamide and Luteolin”: Preliminary results. Eur. Rev. Med. Pharmacol. Sci..

[163] Santhosh L., Block B., Kim S.Y., Raju S., Shah R.J., Thakur N., Brigham E.P., Parker A.M. (2021). Rapid Design and Implementation of Post-COVID-19 Clinics. Chest.

[164] Morrow A.K., Ng R., Vargas G., Jashar D.T., Henning E., Stinson N., Malone L.A. (2021). Postacute/Long COVID in Pediatrics: Development of a Multidisciplinary Rehabilitation Clinic and Preliminary Case Series. Am. J. Phys. Med. Rehabil..

